# An Alternative Preparation of 1-(*N*,*N*-Dimethylaminomethyl)-1′-(diphenylphosphanyl)ferrocene: Synthesis and Structural Characterization of Au^I^ and Pd^II^ Complexes with this Hybrid Ligand

**DOI:** 10.1002/open.201200004

**Published:** 2012-04-12

**Authors:** Petr Štěpnička, Martin Zábranský, Ivana Císařová

**Affiliations:** [a] Department of Inorganic Chemistry, Faculty of Science, Charles University in PragueHlavova 2030, 12840 Prague (Czech Republic) E-mail: stepnic@natur.cuni.cz

**Keywords:** ferrocene ligands, phosphane ligands, hybrid ligands, gold complexes, palladium complexes

## Abstract

1-(*N*,*N*-Dimethylaminomethyl)-1′-(diphenylphosphanyl)ferrocene (**1**) was synthesized in good yield by lithiation of 1-bromo-1′-(diphenylphosphanyl)ferrocene and subsequent reaction with Eschenmoser's salt (dimethylmethylideneammonium iodide). Making use of an easily accessible, nontoxic starting material, this procedure represents a convenient alternative to the original synthetic protocol based on stepwise lithiation/functionalization of 1,1′-bis(tributylstannyl)ferrocene and reductive amination [M. E. Wright, *Organometallics* **1990**, *9*, 853–856]. Compound **1** has typical hybrid-donor properties. When reacted with [AuCl(tht)] (tht=tetrahydrothiophene), it afforded the expected Au^I^ phosphane complex [AuCl(**1**-κP)] (**2**). An attempted removal of the chloride ligand from **2** with AgClO_4_ produced an ill-defined material formulated as Au(**1**)ClO_4_. The uncoordinated amine substituent reacted with traces of hydrogen chloride formed by slow decomposition typically occurring in solution. In this manner, complexes [AuCl(Ph_2_PfcCH_2_NHMe_2_)]Cl (**3**, fc=ferrocene-1,1′-diyl) and [AuCl(Ph_2_PfcCH_2_NHMe_2_)]ClO_4_ (**4**) were isolated from crystallizations experiments with **2** and Au(**1**)ClO_4_, respectively. On a larger scale, complex **3** was prepared easily from **2** and hydrogen chloride. The course of reactions between [PdCl_2_(cod)] (cod=cycloocta-1,5-diene) and **1** were found to depend on the ligand-to-metal ratio. Whereas the reaction with two equivalents of **1** afforded bis(phosphane) complex *trans*-[PdCl_2_(**1**-κP)_2_] (**5**), that of a Pd:P ratio 1:1 produced ligand-bridged dimer [(μ-**1**)PdCl_2_]_2_ (**6**). With hydrogen chloride, complex **6** reacted to afford zwitterionic complex [PdCl_3_(**1**H-κP)] (**7**), which was also formed when ligand **1** and [PdCl_2_(cod)] were allowed to react slowly by liquid-phase diffusion of their chloroform solutions. The compounds were characterized by spectroscopic methods (multinuclear NMR and ESI–MS), and the molecular structures of complex **2**–**4**, **6**⋅2CHCl_3_ and **7**⋅1.5CHCl_3_ were determined by single-crystal X-ray diffraction analysis.

## Introduction

**Scheme 1:**
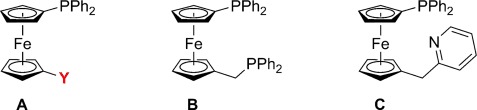
Modified phosphinoferrocene ligands, where Y is a functional group.

**Scheme 2:**
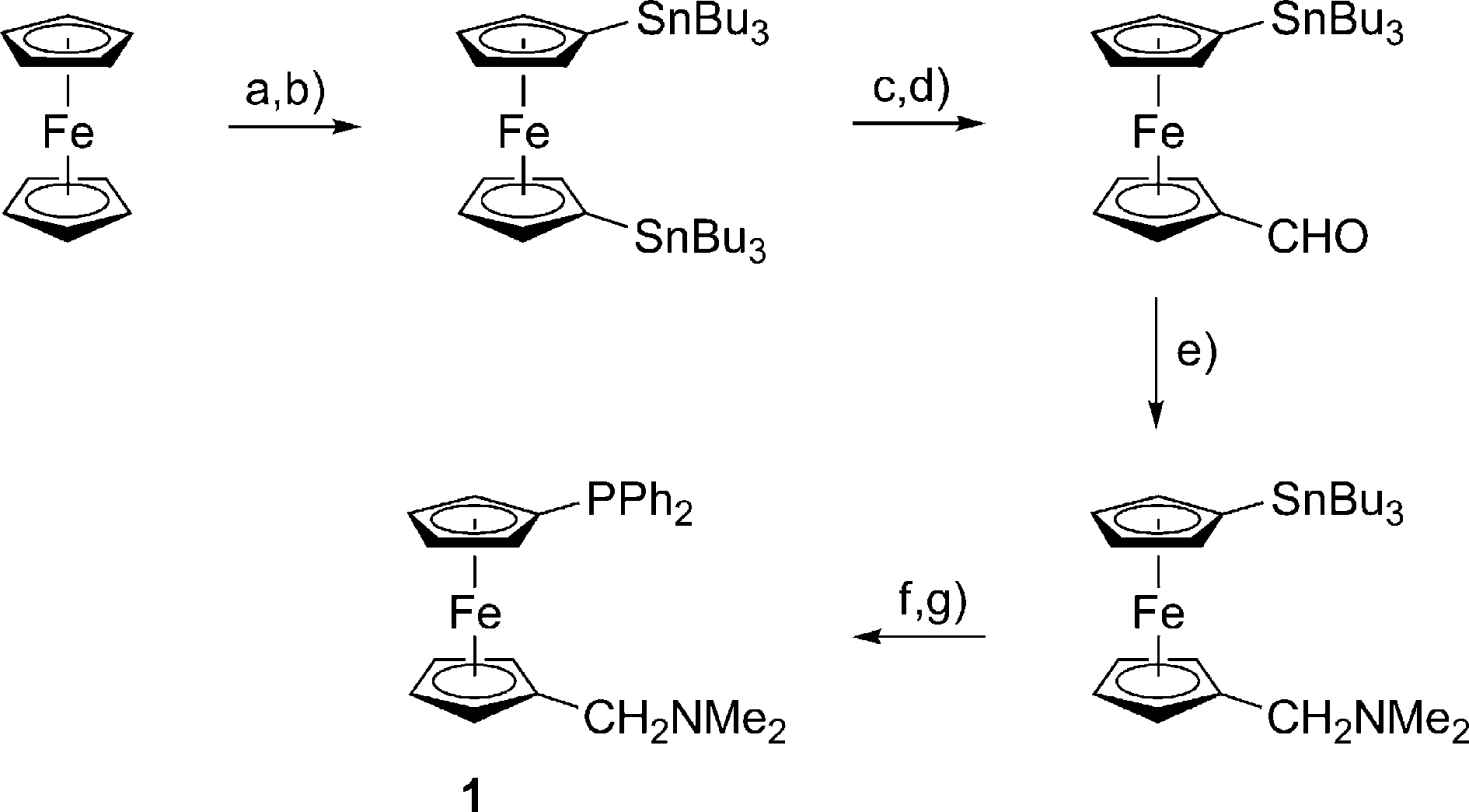
The original preparation of phosphanylamine 1.[8] *Reagents and conditions*: a) LiBu/TMEDA, b) Bu_3_SnCl, c) LiBu, d) DMF, e) Me_2_NH/NaBH_3_(CN), f) LiBu, g) Ph_2_PCl. TMEDA=*N*,*N*,*N*′,*N*′-tetramethyl-1,2-diaminoethane.

**Scheme 3:**
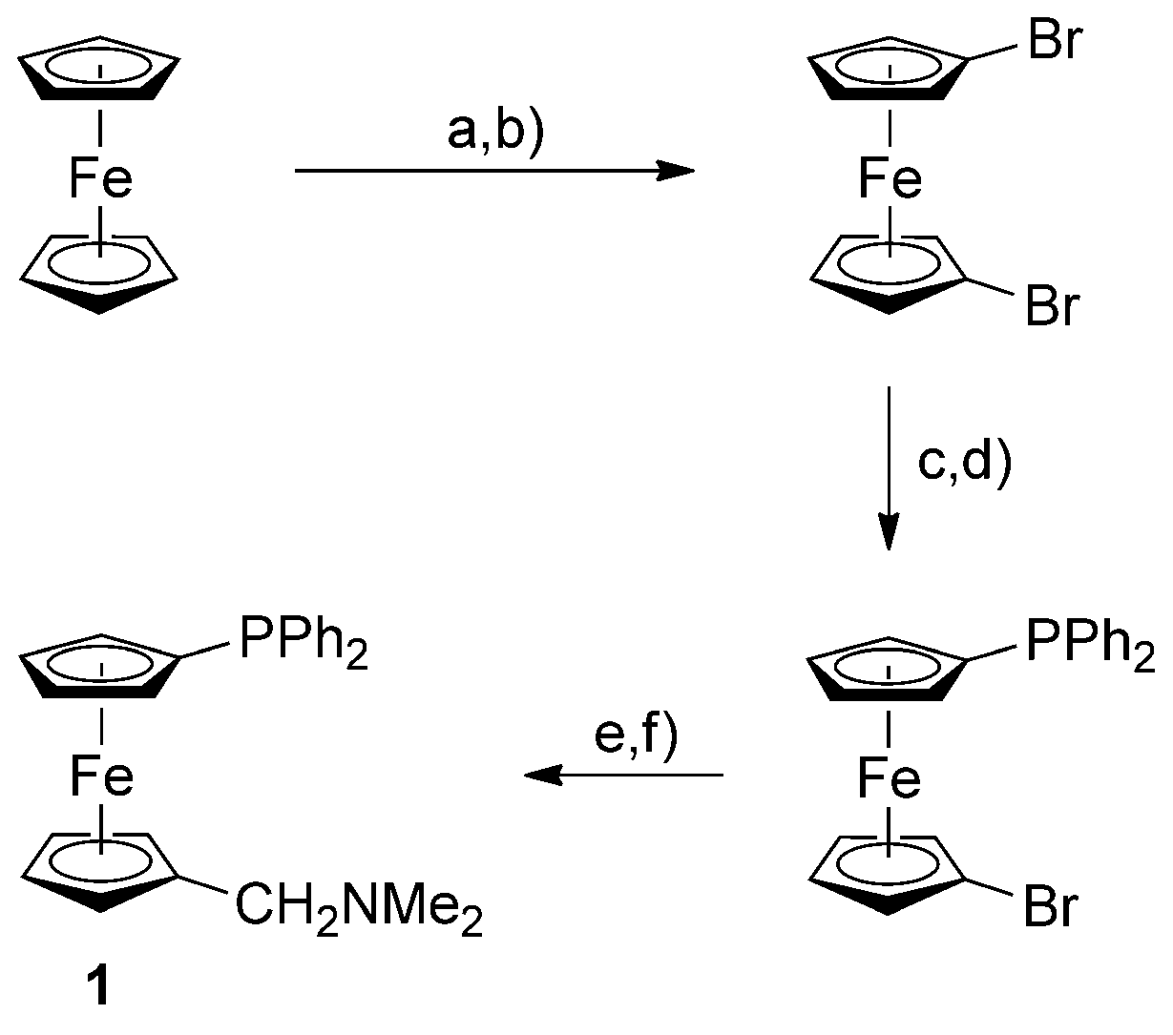
Modified synthesis of phospanylamine 1. *Reagents and conditions*: a) LiBu/TMEDA, b) 1,2-dibromotetrafluoroethane (or an alternative brominating agent), c) LiBu, d) Ph_2_PCl; e) LiBu, f) [CH_2_=NMe_2_]I. TMEDA: *N*,*N*,*N*′,*N*′-tetramethyl-1,2-diaminoethane.

Nowadays, phosphanylferrocene ligands are indispensable in coordination chemistry and homogeneous catalysis.[1] Although a vast number of various simple and chiral ferrocene phosphanes have been designed, prepared and tested to date, distinct areas for further development still remain. Typical examples of “whitespace” in the chemistry of ferrocene donors are simple phosphanylferrocene ligands bearing an additional functional group, most commonly a donor, at position 1’ (**A** in Scheme 1),[2, 3] representing counterparts of the well-studied, donor-symmetric ligand, 1,1′-bis(diphenylphosphanyl)ferrocene (dppf).[4]

In order to expand the family of such modified ferrocene phosphanes, we recently turned to phosphanylferrocene ligands in which the additional donor group is separated from the ferrocene unit by a methylene spacer. So far, we have synthesized and explored the coordination and catalytic properties of an unsymmetric diphosphane Ph_2_PfcCH_2_PPh_2_ (**B**)[5] and related phosphanylpyridine Ph_2_PfcCH_2_Py (**C**) shown in Scheme 1.[6] These compounds are homologues of known ligands, namely the ubiquitous dppf[4] and Ph_2_PfcPy.[7] As a continuation of our work, we decided to study yet another phosphanylferrocene donor of this kind, 1-(*N*,*N*-dimethylaminomethyl)-1′-(diphenylphosphanyl)ferrocene (**1)**. This potentially interesting hybrid P,N-donor, reported more than a decade ago,[8] has not yet been studied as a ligand for transition metal complexes. Herein, we describe an alternative synthesis of compound **1** and investigations into its coordination behavior toward Au^I^ and Pd^II^.

## Results and Discussion

### Alternative preparation of phosphanylamine 1

In his note to *Organometallics* in 1990,[8] Wright reported a four-step synthesis of **1** from ferrocene based on stepwise transmetalation/functionalisation of 1,1′-bis(tributylstannyl)ferrocene[9] and subsequent reductive amination of an intermediate phosphanylaldehyde (Scheme 2). The related compound, Ph_2_PfcCH(Me)NMe_2_, was obtained via nucleophilic opening with phenyllithium of a phosphaferrocenophane substituted with a 1-(*N*,*N*-dimethylamino)ethyl group in position 2 of the ferrocene unit, and subsequent hydrolysis of the reaction mixture.[10]

Our modified procedure also takes advantage of selective transmetalation albeit with 1,1′-dibromoferrocene as the starting material (Scheme 3).[11] This compound, which is commercially available or readily obtained in a one-pot synthesis from ferrocene,11b can be selectively and independently functionalized at position 1 or 1’ and was advantageously used for the preparation of a number of phosphanylferrocene donors.[2, 11] In the present case, 1,1′-dibromoferrocene was firstly lithiated and treated with chlorodiphenylphosphane to give the known stable intermediate 1′-(diphenylphosphanyl)-1-bromoferrocene,11c which was in turn lithiated and reacted with Eschenmoser's salt (dimethylmethylideneammonium iodide)[12, 13] to afford the desired phosphanylamine **1** in a good isolated yield (69 %). (Diphenylphosphanyl)ferrocene as the major side product, resulting from accidental protonation of the lithiated intermediate, was easily removed by chromatography. This newly devised procedure is not only shorter, but also avoids hazardous organotin(IV)-containing reagents and intermediates.

### Synthesis and molecular structures of Au^I^ complexes with 1

**Scheme 4:**
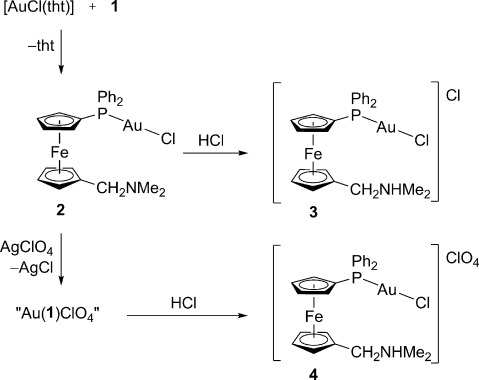
Preparation of Au^I^ complexes 2–4 with ligand 1.

Representing an archetypal example of the so-called hybrid P,N-donors,[14] compound **1** was studied as a ligand for the soft metal ion Au^I^ (Scheme 4). The reaction of **1** with the commonly used Au^I^ precursor [AuCl(tht)] (tht=tetrahydrothiophene) proceeded cleanly to afford the expected phosphane complex **2**. This complex can be isolated by precipitation as a reasonably stable, analytically pure solid. It should be noted, however, that complex **2** as well as other Au^I^ complexes reported in this study decompose slowly when manipulated in solution or exposed to diffuse light. In addition to decomposition, attempts to prepare defined crystalline samples are hampered by the relatively high solubility of these compounds and their general reluctance to crystallize.

The ^1^H NMR spectrum of complex **2** displays resonances due to the 1,1′-disubstituted ferrocene moiety and the attached substituents. In comparison with uncoordinated **1**, the signals of the CH_2_NMe_2_ group are seen at higher fields, while the ferrocene resonances appear shifted to lower fields. The ^31^P NMR signal gives a singlet at *δ*_P_=29.0, shifted by approximately 46 ppm to lower fields versus free **1**. ESI-MS corroborates the formulation of **2** by showing abundant ions at *m*/*z*=660.0 corresponding to protonated **2** (i.e., [AuCl(**1**)+H]^+^).

Attempts to prepare a defined Au^I^ complex in which **1** acts as a P,N-donor group failed. The removal of the Au-bound chloride with AgClO_4_ only afforded a yellow amorphous solid tentatively formulated as Au(**1**)ClO_4_. Other soluble Ag^I^ salts behaved similarly. Although the exact nature of Au(**1**)ClO_4_ might be rather complicated, the material displays only one set of broad signals in its ^1^H NMR spectrum and a single ^31^P NMR resonance at *δ*_P_=41.9, which is shifted 13 ppm downfield relative to the resonance of **2**. The ESI-MS of Au(**1**)ClO_4_ is rather inconclusive, showing signals at *m*/*z*=1051.2 ([Au(**1**)_2_]^+^, the heaviest fragment ion observed), 624.2 ([Au(**1**)]^+^), 503.7 ([Au(**1**)Ph_2_PfcCH_2_]^2+^) and 383.2 ([Ph_2_PfcCH_2_]^+^).

Repeated crystallization experiments with **2** in various solvents were unsuccessful, affording only dark intractable oils. Finally, a small amount of yellow crystalline material was isolated from an ethyl acetate/hexane mixture and structurally characterized as the hydrochloride of **3**. Very likely, decomposition in solution produced traces of hydrogen chloride, which in turn reacted with the free amine group to give less soluble salt **3**. Complex **3** was later prepared on a larger scale by protonation of **2** with hydrogen chloride in a slight excess in methanolic solution. Analogous complex **4** was isolated from crystallization experiments with Au(**1**)ClO_4_ (generated in situ) from dichloromethane/toluene. In this case, the halogenated solvent was the probable source of hydrogen chloride.

Protonation at the amine group is clearly indicated in the ^1^H and ^13^C NMR spectra. The ^1^H NMR resonances of **3** appeared at lower fields compared with **2** (Δ*δ*_H_=+0.47 for NCH_3_ and Δ*δ*_H_=+0.93 ppm for NCH_2_), while the ^13^C NMR signals were shifted to higher fields (Δ*δ*_c_≍−2.2 for NCH_3_ and Δ*δ*_c_≍−1.4 ppm for NCH_2_). In contrast, the ^31^P NMR signal of **3** was observed at a similar *δ* value as **2** and the ESI-MS displayed only the signal arising from [AuCl(**1**)+H]^+^ at *m*/*z*=660.0.

**Figure 1:**
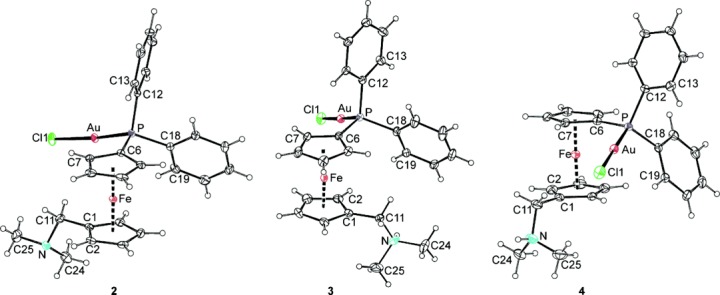
Views of the molecular structures of complex 2 and cationic compounds 3 and 4. Displacement ellipsoids enclose the 30 % probability level. For clarity, only one position of the disordered NMe_2_ group is shown for the cation in compound 3.

The molecular structures of complexes **2**–**4** were determined by single-crystal diffraction analysis and are presented in Figure 1. Selected geometric data are listed in Table 1. The Cl–Au–P angles found in complexes **2**–**4** are close to 180°, a value expected for Au^I^, while the Au–donor distances compare well with those reported earlier for [(μ-dppf)(AuCl)_2_][15] and [AuCl(FcPPh_2_-κP)] (Fc=ferrocenyl).[16] The ferrocene units in **2**–**4** show regular geometries with marginal variation in the Fe–C distances (≤0.035 Å for the individual compounds) and tilt angles (∢Cp1,Cp2) not exceeding 4°. The substituents at the ferrocene unit assume practically ideal synclinal eclipsed conformations in **2** and **3** (ideal value: *τ*=72°), whereas in **4**, they are more distant, adopting an intermediate conformation (*τ*=93°; see Table 1).

**Table 1:** Selected distances [Å] and angles [°] for complexes **2–4**

Parameter	**2**	**3**[d]	**4**
Au–Cl1	2.2925(9)	2.2957(8)	2.286(1)
Au–P	2.2223(8)	2.2281(8)	2.232(1)
∢P–Au–Cl1	171.45(3)	177.94(3)	179.44(5)
Fe–Cg1[a]	1.652(2)	1.647(2)	1.647(2)
Fe–Cg2[a]	1.645(2)	1.643(2)	1.647(2)
∢Cp1,Cp2[b]	2.2(2)	3.3(2)	4.0(3)
*τ*[c]	74	71	93
C6–P	1.778(3)	1.784(3)	1.789(5)
C1–C11	1.508(5)	1.483(4)	1.489(7)
C11–N	1.482(4)	1.488(5)	1.517(6)
∢C1–C11–N	112.9(3)	113.7(3)	112.3(4)

[a] Cg1 and Cg2 are the centroids of the cyclopentadienyl rings Cp 1 and Cp 2, respectively.

[b] Cp1=C(1–5), Cp2=C(6–10).

[c] *τ*=torsion angle C1–Cg1–Cg2–C6.

[d] Values for the major contributing part in the disordered moiety are given.

In all three molecules, one of the phenyl groups points above the ferrocene unit, while the other and the AuCl moiety are directed below the PPh_2_-substituted cyclopentadienyl ring (i.e., toward the iron atom). The CH_2_NMe_2_ groups are directed away from the ferrocene moiety (cf., the torsion angles C2–C1–C11–N: 81.4(4)° for **2**, 71.4(5)° for **3**, and 96.1(6)° for **4**). Their protonation (such in **3** and **4**) results in a slight elongation of the N–Me and N–C11 bonds and shortening of the C1–C11 distance compared with the structure of the parent compound **2**.

**Figure 2:**
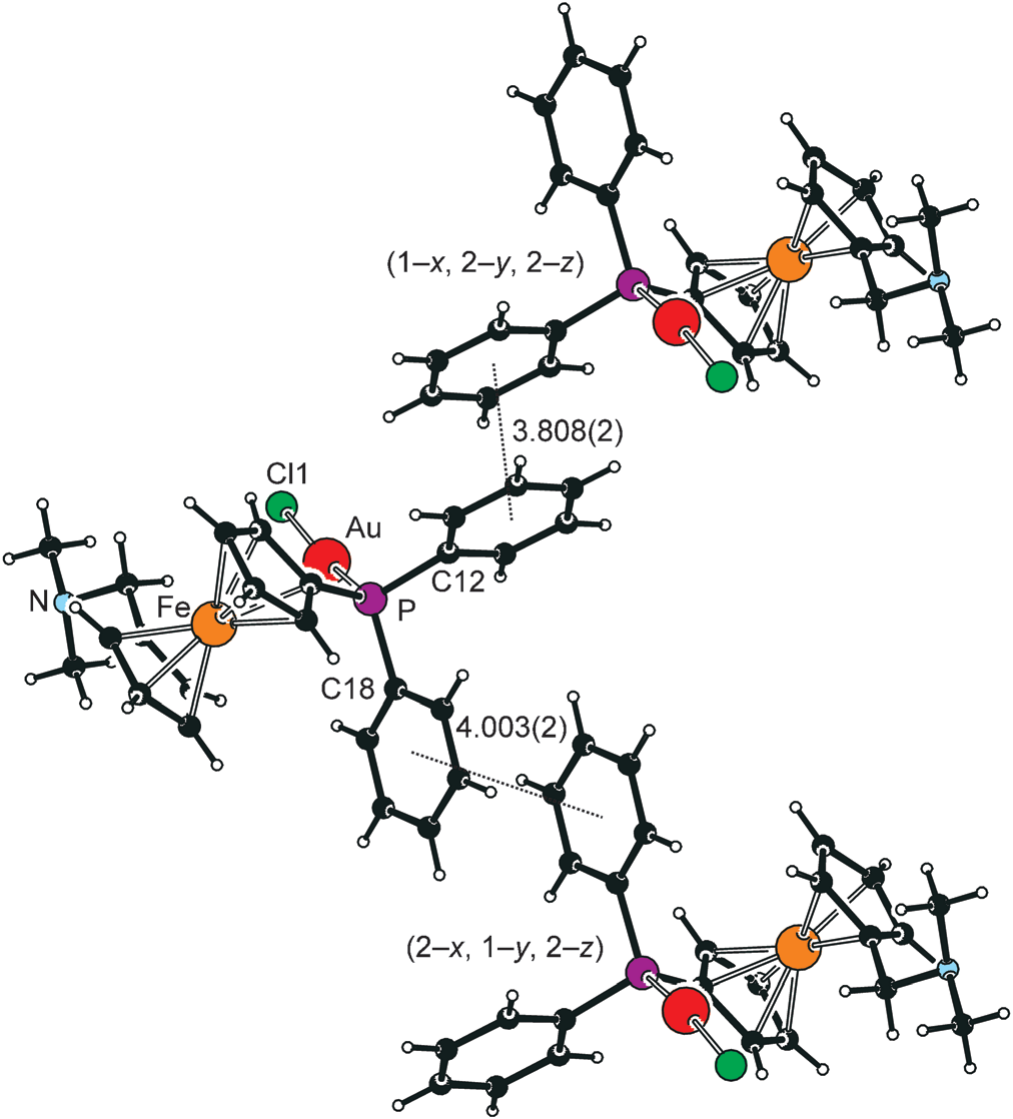
π⋅⋅⋅π Stacking interactions of inversion-related molecules (••••) in the crystal structure of 2. Distances between the centroids of the interacting rings [Å] and symmetry codes are given.

**Figure 3:**
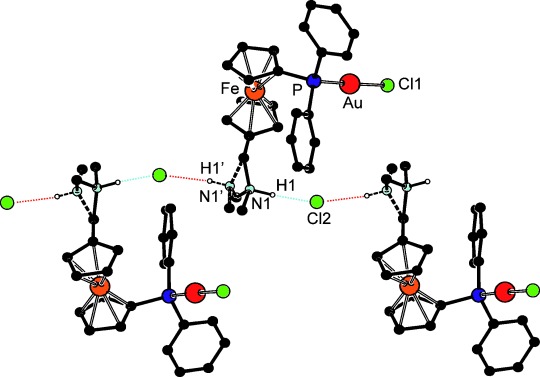
Hydrogen-bonding interactions in the crystal structure of compound 3. Each NH group forms one N–H⋅⋅⋅Cl contact (major (••••) and minor (••••) contributing orientation of the disordered CH_2_NHMe_2_ moiety). Hydrogen bond parameters are as follows: N1⋅⋅⋅Cl2=3.075(4) Å, angle at H1=172°; N1′⋅⋅⋅Cl2=3.20(1) Å, angle at H1′=172°.

No significant intermolecular Au⋅⋅⋅Au (aurophilic) contacts[17] were detected in the structures studied. Individual molecules in the crystal of **2** associate into infinite zig-zag chains via π–π stacking interactions of inversion-related (i.e., exactly parallel) benzene rings (Figure 2). These chains are interlinked via C–H⋅⋅⋅Cl interactions. Protonation of the amine nitrogen introduces an NH proton into the structure that is suitable for the formation of charge-assisted hydrogen bonds. As a result, the ions constituting the crystals of **3** assemble via N1–H1⋅⋅⋅Cl2 hydrogen bonds (Figure 3) with each CH_2_NMe_2_H group interacting with one proximal chloride ion. These interactions result in a discrete (nonpolymeric) regular array of ion pairs with two alternative orientations due to disorder. Similarly to **3**, the crystal packing of **4** is dominated by hydrogen-bonding interactions of the perchlorate oxygens with the NH proton (N⋅⋅⋅O: 3.115(7) and 2.944(9) Å for two perchlorate oxygens O1 and O2), which operate together with soft intermolecular C–H⋅⋅⋅O contacts.

### Synthesis and molecular structures of Pd^II^ complexes with 1

**Scheme 5:**
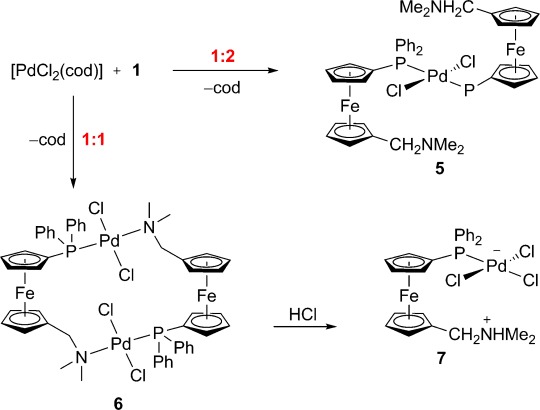
Preparation of Pd^II^ complexes 5–7 with ligand 1.

The preparation of Pd^II^ complexes from ligand **1** are summarized in Scheme 5. As expected, two equivalents of the ligand reacted smoothly with [PdCl_2_(cod)] (cod=cycloocta-1,5-diene) to afford *trans*-bis(phosphane) complex **5**. In its ^1^H NMR spectrum, complex **5** showed one set of signals due to coordinated **1**. The ^31^P NMR spectrum was indicative of *trans* geometry, displaying a single resonance at *δ*_P_=15.7 ppm, which is close to chemical shifts observed for structurally characterized complexes of the type *trans*-[PdCl_2_(Ph_2_PfcX-κ*P*)_2_], where X=P(O)Ph_2_,[18] Py, CH_2_Py,[19] CH=CH_2_,[20] PO_3_Et_2_,[21] SMe,[22] CO_2_H,[23] CONHPh,[24] CONHY (Y=H or NH_2_),[25] CONH(CH_2_)_2_Py,[26] CONHCH_2_COY (Y=OH or NH_2_),[27] or CONH_2−*n*_(CH_2_CH_2_OH)_*n*_ (*n*=1, 2).[28]

Lowering the ligand-to-metal molar ratio to 1:1 resulted in the formation of a poorly soluble material showing broad ^1^H NMR signals and a single resonance in the ^31^P NMR spectrum at *δ*_P_=23.3 ppm. Considering the results of the crystallographic study (see below), this compound was formulated as symmetric dimer **6**. A subsequent reaction of **6** with hydrogen chloride produced zwitterionic complex **7**. Similarly to the Au^I^ complexes discussed above, the protonation of the CH_2_NMe_2_ groups was clearly manifested in the ^1^H NMR spectrum, while the signal in the ^31^P NMR of **7** was observed at a position similar to that of **6**.

Crystallization experiments further demonstrated the hemilabile nature of **1**. For instance, crystals of the solvate **6**⋅2CHCl_3_ were isolated upon recrystallization of the residue obtained by evaporation of the mother liquor remaining after isolation of **5**. Moreover, an attempted crystallization by liquid-phase diffusion of chloroform solutions of ligand **1** and [PdCl_2_(cod)] (equimolar amounts) produced orange-red crystals of **7**⋅1.5CHCl_3_. Apparently, the basic amine group (either free or released from the coordination sphere of Pd^II^) became protonated in the presence of hydrogen chloride that is formed by the slow decomposition of the halogenated solvent or added intentionally (cf. preparation of **7** from **6** in the Experimental Section).

**Figure 4:**
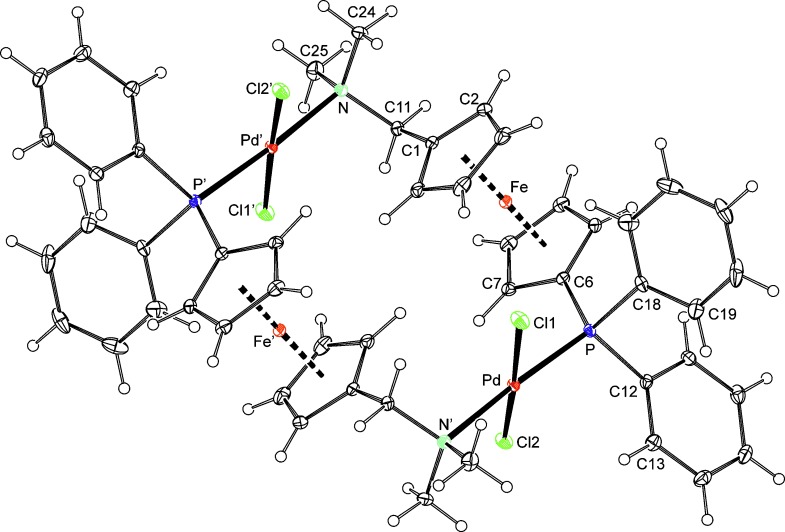
View of the molecular structure of complex 6⋅2CHCl3. Displacement ellipsoids are scaled to the 30 % probability level. Selected distances [Å]: Pd–Cl1 2.2947(5), Pd–Cl2 2.3099(5), Pd–P 2.2527(5), Pd–N’ 2.211(2), Fe–Cg1 1.6630(9), Fe–Cg2 1.6568(9), C6–P 1.799(2), C1–C11 1.489(3), C11–N 1.498(2); selected angles [°]: Cl1–Pd–P 89.45(2), Cl1–Pd–N’ 89.87(4), Cl2–Pd–P 87.74(2), Cl2–Pd–N’ 93.32(4), C1–C11–N 111.4(2); Cg1 and Cg2 are the centroids of the cyclopentadienyl rings C(1–5) and C(6–10), respectively.

**Figure 5:**
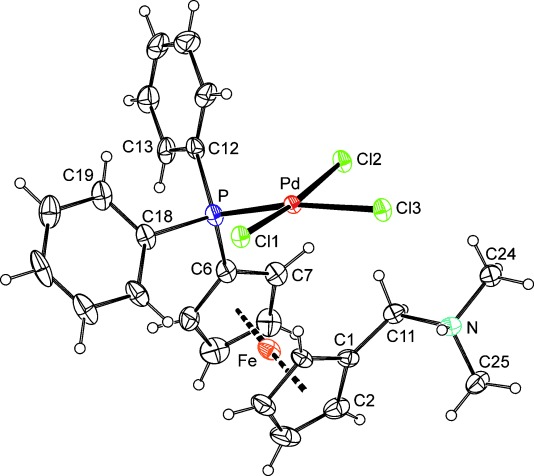
View of the molecular structure of complex 7⋅1.5CHCl_3_ showing the atom labeling scheme and displacement ellipsoids at the 30 % probability level. Selected distances [Å]: Pd–P 2.237(2), Pd–Cl1 2.303(2), Pd–Cl2 2.317(2), Pd–Cl3 2.390(2), Fe–Cg1 1.652(5), Fe–Cg2 1.655(5), C6–P 1.80(1), C1–C11 1.47(1), C11–N 1.50(1); selected angles [°]: Cl1–Pd–Cl3 90.71(7), Cl1–Pd–P 91.66(8), Cl2–Pd–Cl3 89.90(7), Cl2–Pd–P 88.93(8), C1–C11–N 114.7(7); Cg1 and Cg2 are the centroids of the cyclopentadienyl rings C(1–5) and C(6–10), respectively.

The crystal structures of **6**⋅2CHCl_3_ and **7**⋅1.5CHCl_3_ along with relevant geometric data are presented in Figures 4 and 5, respectively. It is noteworthy that, according to a search of The Cambridge Structural Database,[29] structurally characterized bis(dichloridopalladium) complexes symmetrically bridged by two P,N donors are limited to only several compounds that are obtained from N-heterocyclic ligands bearing phosphane substituents.[30] Indeed, the coordination geometry of characterized compound **6**⋅2CHCl_3_ is quite similar to those found in similar dimers prepared from ligands Ph_2_PCH_2_CH_2_Py30c, [31] and 2-Ph_2_PCH_2_O(CH_2_)_3_Py.30a

In complex **6**, Pd and its four ligating atoms are coplanar within ∼0.1 Å, and the interligand angles deviate from the ideal 90° by less than ∼3°. This corresponds with the sum of the interligand angles of 360.4°, which in turn rules out any significant tetrahedral distortion. The ferrocene moiety adopts an intermediate conformation with a *τ* value of 157°, which makes the ferrocene substituents more distant in **6** than in Au^I^ complexes discussed above. The ferrocene cyclopentadienyl groups are mutually tilted by 6.8(1)°. The coordinated CH_2_NMe_2_ group is directed away from the ferrocene unit (C5–C1–C11–N=80.5(2)°), in a similar way to the free or protonated CH_2_NMe_2_ moiety in the Au^I^ complexes.

The coordination geometry in complex **7** (see Figure 5) compares well with that reported for the anion in [PhCH_2_NHMe_2_][PdCl_3_(PPh_3_)][32] and in the zwitterionic Fe-Pd-Ni complex [Cl_3_Pd(μ-Ph_2_Pfctpy)NiCl(DMSO)_2_] (tpy=2,2′:6,2′′-terpyridin-4-yl, DMSO=dimethyl sulfoxide).[33] In accordance with a larger *trans* influence of the phosphane donor,[34] the Pd–Cl bond *trans* to the phosphorus atom is significantly longer than the two remaining ones. The coordination environment of Pd in complex **7** is more distorted than in **6**, with the maximum deviation from the mean PdCl_3_P plane being approximately 0.19 Å. Accordingly, the sum of the interligand angles in **7**⋅1.5CHCl_3_ (361.2°) exceeds the value expected for an ideally square-planar coordination (360°).

The cyclopentadienyl rings in **7** are tilted by 5.3(6)° and assume a practically ideal synclinal eclipsed conformation (*τ*=74°). The CH_2_NHMe_2_ group is directed to the side of the PdCl_3_P plane and away from the phosphine group, being practically perpendicular to the parent Cp1 plane (C5–C1–C11–N=94(1)°). Individual molecules in the structure of **7**⋅1.5CHCl_3_ associate into centrosymmetric dimers via pairs of N–H1N⋅⋅⋅Cl3 hydrogen bonds (N⋅⋅⋅Cl3=3.170(7) Å, angle at H1N=160°). Additional intermolecular contacts can be detected between the chloride ligands and one of the polarized hydrogen atoms in the NMe_2_ groups and the hydrogens of both solvent molecules.

## Conclusions

An alternative preparation of 1-(*N*,*N*-dimethylaminomethyl)-1′-(diphenylphosphanyl)ferrocene (**1**) was developed, making use of 1-bromo-1′-(diphenylphosphanyl)ferrocene as a convenient nontoxic precursor. Phosphanylamine **1** can be regarded a typical hybrid, potentially hemilabile ligand.[14] When used as a donor for the soft AuCl fragment possessing a single vacant coordination site, **1** coordinates as expected through its soft phosphanyl donor group, while the uncoordinated amine group acts as a scavenger for traces of hydrogen chloride formed by decomposition of the complex or halogenated solvents over extended periods of time. Attempts to enforce N-coordination by removal of the Au-bound chloride ligand with Ag^I^ salts produce only ill-defined materials, from which again “simple” phosphane–AuCl complexes (albeit with protonated amine groups) were isolated, resulting from the reaction with adventitious hydrogen chloride.

With Pd^II^, which is known to be readily coordinated by both P and N donors,[34] compound **1** formed the expected *trans*bis(phosphane) complex at a ligand-to-metal ratio of 2:1 (the soft phosphane group is preferentially coordinated). A similar reaction but at a 1:1 ratio of metal to ligand afforded the symmetric, ligand-bridged dimer [(μ-**1**)PdCl_2_]_2_, which was readily cleaved with hydrogen chloride to afford the zwitterionic complex [PdCl_3_(**1**H-κ*P*)]. Such behavior again underscores the hybrid nature of phosphanylamine **1**.

## Experimental Section

### Synthesis

**General**: The syntheses were performed in argon-flushed vessels and, in the case of Au^I^ complexes, also under exclusion of direct day light. Ph_2_PfcBr11c and [AuCl(tht)][35] were prepared according to literature methods. THF and MeOH were distilled from sodium/benzophenone ketyl and NaOMe, respectively. Dry CH_2_Cl_2_ (Sigma–Aldrich) as well as other solvents (Lach-Ner, Czech Republic) were used as received. ^1^H NMR spectra were recorded at 298 K on a UNITY Inova 400 spectrometer (Varian, Palo Alto, CA, USA). Chemical shifts (*δ*/ppm) are given relative to internal tetramethylsilane (*δ*_H_=*δ*_C_=0) or to external 85 % aq H_3_PO_4_ (*δ*_P_=0). In addition to the usual notation of the multiplicity of NMR signals, virtual triplets (vt) and virtual quartets (vq) arising from AA′BB’ and AA′BB′X spin systems of the ferrocene cyclopentadienyl groups (A, B=^1^H, X=^31^P) are indicated. ESI-MS were recorded using an Esquire 3000 spectrometer (Bruker, Billerica, MA, USA) in methanolic solutions. The identities of the observed ionic species were confirmed by comparison of theoretical and experimentally determined isotopic patterns. The Pd^II^ complexes could not be analyzed by ESI-MS due to a very low intensity of the higher mass signals. **CAUTION!** Although we have not encountered any problems, it should be noted that perchlorate salts of metal complexes with organic ligands are potentially explosive and, therefore, should be handled with care and only in small quantities.

**1-(*N***,***N*****-Dimethylaminomethyl)-1′-(diphenylphosphanyl)ferrocene (1)**: An oven-dried 100 mL Schlenk flask was charged with Ph_2_PfcBr (2.02 g, 4.5 mmol) and connected with a bent glass tube (120° elbow) to another 100 mL Schlenk flask containing solid [CH_2_=NMe_2_]I (Eschenmoser's salt, 1.39 g, 7.5 mmol). The reaction vessel was carefully flushed with argon before dry THF (60 mL) was introduced to the Ph_2_PfcBr-containing flask. The resulting solution was cooled in a dry ice/EtOH bath. After LiBu (2.4 mL, 2.5 m in hexane, 6 mmol) was added, the reaction mixture was stirred for 15 min at −78 °C. Next, the Ph_2_PfcLi-containing reaction mixture was poured onto pre-cooled (−78 °C) [CH_2_=NMe_2_]I via the bent glass tube, and the resulting mixture was stirred first at −78 °C for 15 min and then at RT overnight. The mixture was quenched by addition of saturated aq NaHCO_3_ (10 mL) and extracted several times with Et_2_O. The combined organic extracts were washed with brine, dried over MgSO_4_, filtered and evaporated in vacuo leaving an orange residue, which was purified by column chromatography over alumina. The column was washed first with CH_2_Cl_2_ to remove (diphenylphosphanyl)ferrocene and then with CH_2_Cl_2_/MeOH (10:1 v/v) to elute crude amine **1**. The solvents were evaporated in vacuo, and crude amine **1** was purified over a silica gel column (CH_2_Cl_2_/MeOH/NEt_3_, 20:1:1 v/v). Evaporation of the first band afforded analytically pure **1** as a yellow-orange solid (1.32 g, 69 %): ^1^H NMR (400 MHz, CDCl_3_): *δ*=2.10 (s, 6 H, CH_2_N*Me*_2_), 3.00 (s, 2 H, C*H*_2_NMe_2_), 4.02 (vt, *J*′=1.9 Hz, 2 H, fc), 4.04 (vq, *J*′=1.9 Hz, 2 H, fc), 4.10 (vt, *J*′=1.8 Hz, 2 H, fc), 4.32 (vt, *J*′=1.8 Hz, 2 H, fc), 7.28–7.41 ppm (m, 10 H, PPh_2_); ^31^P NMR (162 MHz, CDCl_3_): *δ*=−16.2 ppm; ^13^C NMR (101 MHz, CDCl_3_): *δ*=44.6 (CH_2_N*Me*_2_), 58.5 (*C*H_2_NMe_2_), 69.3 (*C*H of fc), 71.3 (*C*H of fc), 71.3 (d, *J*_P−C_=4 Hz, *C*H of fc), 73.4 (d, *J*_P−C_=15 Hz, *C*H of fc), 76.0 (d, ^1^*J*_P−C_=9 Hz, *C*−P of fc), 83.9 (*C*–CH_2_ of fc), 128.1 (d, ^3^*J*_P−C_=7 Hz, *C*H_*meta*_ of PPh_2_), 128.5 (*C*H_*para*_ of PPh_2_), 133.5 (d, ^2^*J*_P−C_=20 Hz, *C*H_*ortho*_ of PPh_2_), 139.2 ppm (d, ^1^*J*_P−C_=10 Hz, *C_ipso_* of PPh_2_). The NMR data correspond to the literature, except for the ^31^P NMR chemical shift, which was reported to be 11.8 ppm in the original paper.[8]

**Chlorido[1-(*N***,***N*****-dimethylaminomethyl)-1′-(diphenylphosphanyl-κ*P*)ferrocene]gold(I) (2)**: A solution of compound **1** (215 mg, 0.50 mmol) in CH_2_Cl_2_ (2 mL) was added to a solution of [AuCl(tht)] (160 mg, 0.50 mmol) in CH_2_Cl_2_ (2 mL). The mixture was stirred in the dark at RT for 30 min and then filtered into pentane (200 mL). The solution was allowed to stand at −18 °C overnight, and the resulting precipitate was filtered, washed with cold pentane and carefully dried in vacuo to give complex **2** as a yellow solid (276 mg, 84 %): ^1^H NMR (400 MHz, CDCl_3_): *δ*=2.27 (s, 6 H, CH_2_N*Me*_2_), 3.36 (s, 2 H, C*H*_2_NMe_2_), 4.14 (vt, *J*′=1.9 Hz, 2 H, fc), 4.28 (dvt, *J*=3.0, 1.9 Hz, 2 H, fc), 4.39 (vt, *J*′=1.9 Hz, 2 H, fc), 4.57 (dvt, *J*=1.1, 1.9 Hz, 2 H, fc), 7.43–7.61 ppm (m, 10 H, PPh_*2*_); ^31^P NMR (162 MHz, CDCl_3_): *δ*=29.0 ppm; ^13^C NMR (101 MHz, CDCl_3_): *δ*=43.9 (CH_2_N*Me*_2_), 57.6 (*C*H_2_NMe_2_), 69.5 (d, ^1^*J*_P−C_=73 Hz, *C*–P of fc), 70.5 (*C*H of fc), 73.0 (*C*H of fc), 73.1 (d, *J*_P−C_=9 Hz, *C*H of fc), 74.1 (d, *J*_P−C_=14 Hz, *C*H of fc), 82.9 (*C*–CH_2_ of fc,), 129.0 (d, ^3^*J*_P−C_=12 Hz, *C*H_*meta*_ of PPh_2_), 130.6 (d, ^1^*J*_P−C_=63 Hz, *C_ipso_* of PPh_2_), 131.8 (d, ^4^*J*_P−C_=2 Hz, *C*H_*para*_ of PPh_2_), 133.5 ppm (d, ^2^*J*_P−C_=13 Hz, *C*H_*ortho*_ of PPh_2_); MS (ESI): *m*/*z*: 660.0 [*M*+H]^+^; Anal. calcd for C_25_H_26_AuClFeNP: C 45.51, H 3.97, N 2.12, found: C 45.53, H 4.10, N 2.04.

**Chlorido{dimethyl[1′-(diphenylphosphanyl-κ*P*)-ferrocen-1-yl)methyl]ammonium}gold(I) chloride (3)**: Methanolic HCl (0.18 mL, 0.695 m, 0.125 mmol) was added to a suspension of complex **2** (66.5 mg, 0.10 mmol) in dry MeOH (3 mL). The solid complex dissolved to give a clear orange solution, which was stirred in the dark for 1 h and then precipitated with Et_2_O (50 mL). After standing at −18 °C overnight, the precipitated product was filtered, washed with cold Et_2_O and pentane and dried in vacuo to give **3** as a yellow solid (36.5 mg, 52 %). Complex **3** was also obtained upon prolonged crystallization of **1** from EtOAc/hexane: ^1^H NMR (400 MHz, CDCl_3_): *δ*=2.74 (s, 6 H, CH_2_NH*Me*_2_), 4.17 (br vt, *J*’≍1.8 Hz, 2 H, fc), 4.25 (br m, 2 H, fc), 4.29 (br s, 2 H, C*H*_2_NHMe_2_), 4.68 (br m, 2 H, fc), 4.81 (br vt, *J*’≍1.8 Hz, 2 H, fc), 7.45–7.60 ppm (m, 10 H, PPh_*2*_); ^31^P NMR (162 MHz, CDCl_3_): *δ*=28.7 ppm; ^13^C NMR (101 MHz, CDCl_3_): *δ*=41.7 (CH_2_NH*Me*_2_), 56.2 (*C*H_2_NHMe_2_), 70.6 (d, ^1^*J*_P−C_=73 Hz, *C*–P of fc), 71.2 (*C*H of fc), 73.3 (d, *J*_P−C_=9 Hz, *C*H of fc), 74.4 (d, *J*_P−C_=13 Hz, *C*H of fc), 74.8 (*C*H of fc), 76.1 (*C*–CH_2_ of fc), 129.2 (d, ^3^*J*_P−C_=12 Hz, *C*H_*meta*_ of PPh_2_), 129.7 (d, ^1^*J*_P−C_=64 Hz, *C_ipso_* of PPh_2_), 132.1 (d, ^4^*J*_P−C_=3 Hz, *C*H_*para*_ of PPh_2_), 133.4 ppm (d, ^2^*J*_P−C_=13 Hz, *C*H_*ortho*_ of PPh_2_); MS (ESI): *m*/*z*: 660.0 [*M*+H]^+^; Anal. calcd for C_25_H_27_AuCl_2_FeNP: C 43.13, H 3.91, N 2.01, found: C 43.32, H 4.31, N 1.68.

**Chlorido{dimethyl[1′-(diphenylphosphanyl-κ*P*)-ferrocen-1-yl)methyl]ammonium}gold(I) perchlorate (4)**: A solution of silver(I) perchlorate (10.5 mg, 0.05 mmol) in dry THF (1 mL) was added to complex **2** (33 mg, 0.05 mmol) dissolved in CH_2_Cl_2_ (1 mL). The resulting mixture was stirred in the dark for 15 min and filtered through a pad of celite. The filtrate was evaporated in vacuo to afford Au(**1**)ClO_4_ as a yellow solid, which was then redissolved in CH_2_Cl_2_ (ca. 1 mL). The solution was layered with toluene (ca. 1 mL) and allowed to crystallize at +4 °C over several weeks to afford **4** as yellow-orange plates (yield not determined): Anal. calcd for C_25_H_27_AuCl_2_FeNO_4_P: C 39.50, H 3.58, N 1.84, found: C 39.78, H 3.60, N 1.69.

**Dichloridobis[1-(*N***,***N*****-dimethylaminomethyl)-1′-(diphenylphosphanyl-κ*P*)ferrocene]palladium(II) (5)**: A solution of ligand **1** (172 mg, 0.40 mmol) in CH_2_Cl_2_ (2 mL) was added to a suspension of [PdCl_2_(cod)] (57 mg, 0.20 mmol) in CH_2_Cl_2_ (1 mL) whereupon the Pd precursor dissolved to give a clear, dark red solution. After stirring at RT for 3 h, the solution was added drop-wise into Et_2_O (50 mL), and the resulting mixture was allowed to stand at −18 °C overnight. The precipitated product was filtered, washed successively with Et_2_O and pentane and dried in vacuo to give **5** as an orange powder (146 mg, 71 %): ^1^H NMR (400 MHz, CDCl_3_): *δ*=2.24 (s, 6 H, CH_2_N*C*H_3_), 3.40 (s, 2 H, C*H*_2_NCH_3_), 4.36 (br vt, 2 H, fc), 4.39 (vt, *J*’≍1.8 Hz, 2 H, fc), 4.53 (br vt, 2 H, fc), 4.55 (vt, *J*’≍1.8 Hz, 2 H, fc), 7.33–7.69 ppm (m, 10 H, PPh_*2*_); ^31^P NMR (162 MHz, CDCl_3_): *δ*=15.7 ppm; Anal. calcd for C_50_H_52_Cl_2_Fe_2_N_2_P_2_Pd⋅CH_2_Cl_2_: C 54.84, H 4.87, N 2.51, found: C 54.89, H 4.95, N 2.23.

Evaporation of the mother liquor and subsequent crystallization of the residue from CH_2_Cl_2_/Et_2_O afforded red crystals of **6**⋅2CHCl_3_.

**Preparation of “PdCl_2_(1)” (6)**: A solution of ligand **1** (86 mg, 0.20 mmol) in CH_2_Cl_2_ (1 mL) was added to a suspension of [PdCl_2_(cod)] (57 mg, 0.2 mmol) in CH_2_Cl_2_ (2 mL). The starting Pd complex dissolved to afford a clear, deep red solution, which after stirring at RT for 3 h formed an orange precipitate. Et_2_O (50 mL) was added to complete the precipitation, and the mixture was stored at −18 °C overnight. The precipitate was filtered, washed with Et_2_O and pentane, and dried in vacuo to give **6** as a yellow-orange solid (117 mg, 96 %). Complex **6** is very poorly soluble in CDCl_3_ and decomposes when dissolved in [D_6_]DMSO. Its ^1^H NMR spectrum displays broad signals: ^31^P NMR (162 MHz, [D_6_]DMSO): *δ*=23.3 ppm; Anal. calcd for C_25_H_26_Cl_2_FeNPPd⋅H_2_O: C 48.22, H 4.53, N 2.25, found: C 48.51, H 4.25, N 2.04.

Attempts to grow single crystals of **6** by reactive diffusion, that is, by layering a solution of [PdCl_2_(cod)] (14.5 mg, 0.05 mmol) in CHCl_3_ (2 mL) with a solution of **1** (21.5 mg, 0.05 mmol) in CHCl_3_ (0.5 mL) in a 5 mm NMR tube afforded crystals of solvated zwitterionic complex **7**⋅1.5CHCl_3_.

**Preparation of [PdCl_3_(1 H-κ*P*)] (7)**: A solution of phosphanylamine **1** (43 mg, 0.10 mmol) in CH_2_Cl_2_ (2 mL) was added to a stirring suspension of [PdCl_2_(cod)] (28 mg, 0.10 mmol) in CH_2_Cl_2_ (1 mL). The resulting red turbid reaction mixture was treated with an excess of methanolic HCl (0.3 mL, 0.695 m, 0.2 mmol) and stirred for 30 min. Next, the reaction mixture was poured into Et_2_O (30 mL) and allowed to stand at −18 °C overnight. The precipitate was filtered, washed with Et_2_O and pentane, and dried in vacuo to give **7** as an orange-red solid (54 mg, 85 %): ^1^H NMR (400 MHz, [D_6_]DMSO): *δ*=2.79 (s, 6 H, CH_2_NHC*H*_3_), 4.59 (br vq, *J*’≍1.9 Hz, 2 H, fc), 4.66–4.69 (m, 4 H, fc), 4.87 (br s, 2 H, C*H*_2_NHCH_3_), 5.15 (br vt, *J*’≍1.8 Hz, 2 H, fc), 7.37–7.52 (m, 10 H, PPh_*2*_), 9.63 ppm (br s, 1 H, CH_2_N*H*Me_2_); ^31^P NMR (162 MHz, [D_6_]DMSO): *δ*=23.5 ppm; Anal. calcd for C_25_H_27_Cl_3_FeNPPd: C 46.84, H 4.25, N 2.19, found: C 46.88, H 4.25, N 1.96.

### X-ray crystallography

Single crystals suitable for X-ray diffraction analysis were grown by crystallization from CH_2_Cl_2_/THF/hexane for **2** (yellow plate, 0.14×0.21×0.24 mm^3^) and EtOAc/hexane for **3** (orange plate, 0.10×0.39×0.41 mm^3^). The crystal of **4** was selected directly from the reaction batch (yellow plate, 0.04×0.17×0.30 mm^3^). Crystals of the Pd^II^ complexes were obtained as described above (**6**⋅2CHCl_3_: orange plate, 0.13×0.35×0.42 mm^3^; **7**⋅1.5CHCl_3_: red-orange prism, 0.11×0.23×0.36 mm^3^).

The diffraction data (±*h*±*k*±*l*; *θ*_max_=26–27.5°, data completeness ≥ 99.6 %) were collected with an Apex2 diffractometer (Bruker) equipped with a Cryostream Cooler (Oxford Cryosystems, Oxford, UK) using graphite monochromated Mo Kα radiation (*λ*=0.71073 Å). The data were corrected for absorption by using methods included in the diffractometer software. The structures were solved by direct methods and refined by full-matrix least squares routines based on *F*^2^ using SHELXL-97.[36] Hydrogen atoms residing on the nitrogen atoms in **3** and **4** were identified on difference density maps and refined as riding atoms with *U*_iso_(H)=1.2 *U*_eq_(N). The NH proton in the structure of **7**⋅1.5CHCl_3_ was included in its calculated position and assigned with *U*_iso_(H)=1.2 *U*_eq_(N). All CH hydrogen atoms were included in their calculated positions and refined similarly. The terminal NHMe_2_ group in the structure of **3** is disordered and was modeled over two positions with refined occupancies of approximately 1/3 and 2/3.

Relevant crystallographic data and structure refinement parameters are summarized in Table 2 for Au^I^ and Pd^II^ complexes. Geometric data and structural drawings were obtained with a recent version of the PLATON program.[37] All numerical values were rounded with respect to their estimated deviations (esds) given to one decimal place. Parameters relating to atoms in constrained positions (hydrogens) are given without esds.

**Table 2:** Selected crystallographic data, data collection and structure refinement parameters for Au^I^ complexes 2–4 and Pd^II^ complexes 6⋅2CHCl_3_ and 7⋅1.5CHCl_3_[a]

Compound	**2**	**3**	**4**	**6**·2CHCl_3_	**7**·1.5CHCl_3_
Formula	C_25_H_26_AuClFeNP	C_25_H_27_AuCl_2_FeNP	C_25_H_27_AuCl_2_FeNO_4_P	C_52_H_54_Cl_10_Fe_2_N_2_P_2_Pd_2_	C_26.5_H_28.5_Cl_7.5_FeNPPd
*M* [g mol^−1^]	659.70	696.16	760.16	1447.91	820.10
Crystal system	triclinic	monoclinic	triclinic	monoclinic	monoclinic
Space group	*P*-1	*C*2/*c*	*P*-1	*P*2_1_/*c*	*P*2_1_/*c*
*a* [Å]	10.0273(5)	13.5631(3)	9.7117(3)	17.5297(7)	13.0909(4)
*b* [Å]	10.6863(5)	13.9491(3)	9.8221(3)	11.1120(4)	10.8438(3)
*c* [Å]	12.1621(6)	26.8361(5)	14.3476(4)	14.8384(6)	24.3709(8)
*α* [°]	69.880(2)		77.483(1)		
*β* [°]	70.558(2)	95.301(1)	76.974(1)	107.794(1)	90.584(1)
*γ* [°]	79.875(2)		83.712(1)		
*V* [Å^3^], *Z*	1151.2(1), 2	5055.5(2), 8	1299.05(7), 2	2752.1(2), 2	3459.4(2), 4
Diffractions total	14444	36170	22091	51763	30819
*R*_int_ [%][b]	3.29	2.47	3.70	2.73	3.08
Unique/obsd diffrns[c]	5276/4859	5831/5471	5995/5276	6308/5624	6791/5715
*R* (obsd diffrns)[c,d] [%]	2.27	2.26	3.06	2.24	8.29
*R*, w*R* (all data)[d] [%]	2.70, 5.34	2.47, 5.06	3.91, 6.99	2.79/5.21	9.59, 24.0
Δ*ρ* [e Å^−3^]	1.10, −1.13	0.84, −0.71	1.64, −1.36	0.60, −0.64	3.28, −1.00[e]
CCDC entry	865934	865935	865936	870336	870335

[a] *T*=150(2) K.

[b] *R*_int_=Σ|
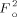
−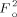
(mean)|/Σ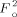, where 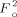
(mean) is the average intensity of symmetry-equivalent diffractions.

[c] Diffractions with *I*_o_>2*σ*(*I*_o_).

[d] *R*=Σ||*F*_o_|−|*F*_c_||/Σ|*F*_o_|, w*R*=[Σ{w(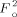
−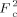)^2^}/Σw(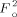)^2^]^1/2^.

[e] Residual electron density in the space accommodating disordered solvent.

CCDC-865934 (**2**), 865935 (**3**), 865936 (**4**), 870336 (**6**⋅2CHCl_3_), and 870335 (**7**⋅1.5CHCl_3_) contain the supplementary crystallographic data for this paper. These data can be obtained free of charge from The Cambridge Crystallographic Data Centre via http://www.ccdc.cam.ac.uk.
